# Recuperating Effect of Sleep

**Published:** 1898-02

**Authors:** 


### Recuperating Effect of Sleep.

Here is an explanation of the recuperating effect of sleep, which it is important for all to consider: Nature takes the time, when one is lying down, to give the heart rest, and that organ consequently makes ten strokes less a minute than when one is in an upright posture. Multiply that by sixty minutes and it is 600 strokes. Therefore in eight hours spent in lying down the heart is saved nearly 5,000 strokes, and as the heart pumps six ounces of blood with each stroke, it lifts 30,000 ounces less of blood in a night of eight hours spent in bed than when one is in an upright position. As the blood flows so much more slowly through the veins when one is lying down extra covering is needed to supply the body with the warmth usually furnished by circulation.—*N. Y. Med. Times.*

